# Impact of Estrogen Therapy on Lymphocyte Homeostasis and the Response to Seasonal Influenza Vaccine in Post-Menopausal Women

**DOI:** 10.1371/journal.pone.0149045

**Published:** 2016-02-09

**Authors:** Flora Engelmann, Andrea Rivera, Byung Park, Marci Messerle-Forbes, Jeffrey T. Jensen, Ilhem Messaoudi

**Affiliations:** 1 Division of Biomedical Sciences, School of Medicine, University of California Riverside, Riverside, California, United States of America; 2 Division of Biostatistics, Department of Public Health and Preventive Medicine, Oregon Health and Science University, Portland, Oregon, United States of America; 3 Department of Obstetrics and Gynecology, Oregon Health and Science University, Portland, Oregon, United States of America; 4 Division of Reproductive Sciences, Oregon National Primate Research Center, Beaverton, Oregon, United States of America; 5 Division of Pathobiology and Immunology, Oregon National Primate Research Center, Beaverton, Oregon, United States of America; Northumbria University, UNITED KINGDOM

## Abstract

It is widely recognized that changes in levels of ovarian steroids modulate severity of autoimmune disease and immune function in young adult women. These observations suggest that the loss of ovarian steroids associated with menopause could affect the age-related decline in immune function, known as immune senescence. Therefore, in this study, we determined the impact of menopause and estrogen therapy (ET) on lymphocyte subset frequency as well as the immune response to seasonal influenza vaccine in three different groups: 1) young adult women (regular menstrual cycles, not on hormonal contraception); 2) post-menopausal (at least 2 years) women who are not receiving any form of hormone therapy (HT) and 3) post-menopausal hysterectomized women receiving ET. Although the numbers of circulating CD4 and CD20 B cells were reduced in the post-menopausal group receiving ET, we also detected a better preservation of naïve B cells, decreased CD4 T cell inflammatory cytokine production, and slightly lower circulating levels of the pro-inflammatory cytokine IL-6. Following vaccination, young adult women generated more robust antibody and T cell responses than both post-menopausal groups. Despite similar vaccine responses between the two post-menopausal groups, we observed a direct correlation between plasma 17β estradiol (E2) levels and fold increase in IgG titers within the ET group. These findings suggest that ET affects immune homeostasis and that higher plasma E2 levels may enhance humoral responses in post-menopausal women.

## Introduction

In addition to their role in sexual differentiation and reproduction, female sex hormones modulate immune function. For instance, β-estradiol (E2) treatment exacerbates the severity of systemic lupus erythematosus and myasthenia gravis [1,2]. On the other hand, the severity and incidence of rheumatoid arthritis and multiple sclerosis are decreased during pregnancy [3] when circulating levels of progesterone are high. Moreover, cytokine production by peripheral blood T cells varies throughout the menstrual cycle. Specifically, the number of PBMC able to secrete IL-4 in response to stimulation correlated with estrogen levels [4] and serum levels of the cytokines IL-6, IL-1β, IL-10, and IL-8 peak during the follicular phase when estrogen levels are highest [5–7]. In addition, vaccination studies in humans indicate that vaginal immunizations are more effective for induction of genital tract antibodies when performed during the mid-follicular phase of the menstrual cycle [8].

The mechanisms by which ovarian steroids affect immune function are beginning to emerge. T and B cells express ERα and ERβ receptors [9] indicating that E2 can directly modulate lymphocyte function. E2 treatment of B cells increases: 1) the expression of the anti-apoptotic molecule Bcl-2 [10–12]; 2) B cell activation [13]; 3) IgG production [14]; and 4) the expression of activation-induced deaminase (AID) [15] leading to increased frequency of somatic hypermutation and class-switch recombination. Similarly, E2 was shown to inhibit activation-induced apoptosis of T cells from lupus patients by down-regulating the expression of Fas ligand [16]. In vitro studies also suggest a potential bias towards Th2, Th17, and Treg polarization in E2 treated T cell cultures [17,18]. Estrogen and progesterone can also indirectly influence T and B cells by affecting the function of innate immune cells such as dendritic cells and macrophages that influence T and B cell differentiation [19]. For example, progesterone treatment reduces the ability of dendritic cells to take up antigenic peptides, stimulate T cell responses [20], and secrete the potent antiviral cytokine IFNα [21]. In contrast, estradiol treatment increases the ability of macrophages to secrete inflammatory cytokines [22].

Aging is associated with a decline in immune function; a phenomenon commonly referred to as immune senescence and believed to result in greater infectious disease related morbidity and mortality in the elderly [23]. Given the influence of ovarian steroids on immune function, their loss during menopause could exacerbate immune senescence [24,25]. This hypothesis is supported by the observation that rhinovirus infection induces a higher IFNγ and IL-13 response in young women than men, however this sex difference is no longer detected after the age of 50 coincident with typical onset of menopause and the associated loss of ovarian steroids [26]. Similarly, hepatitis vaccines induce higher antibody titers and seroconversion rates in young women, but this sexual dimorphism is no longer evident in vaccinees over the age of 60 [27]. A recent study looking at sex differences in gene expression in human peripheral blood found differences between men and women become smaller when women reach menopause and larger when women use hormonal contraceptives [28]. The biological process gene ontology category in female-biased genes with the greatest enrichment was immune system process [28].

Animal studies also support this hypothesis. Ovariectomy of young female rats resulted in decreased leukocyte chemotaxis and LPS-induced proliferation, reduced NK cell lysis, and increased oxidative damage and inflammatory cytokine production by peritoneal macrophages suggestive of premature immunosenescence [29,30]. Studies from our laboratory have shown that ovariectomized female rhesus macaques generate reduced T and B cell responses to vaccination compared to age-matched control animals [31]. Similarly, in a mouse model of HSV-2 challenge, ovariectomy resulted in a reduced response to an experimental vaccine and abrogated protection from vaginal challenge [32].

Additional studies have shown that post-menopausal women have higher plasma levels of the inflammatory cytokines TNFα and IFNγ compared to pre-menopausal subjects [33–35]. In contrast, HT reduces the levels of both of these inflammatory mediators [33,35], in addition to IL-6 [36,37]. Moreover, surgical menopause results in several changes associated with immune senescence such as a decreased CD4/CD8 ratio, an increase in the percentage of NK cells and a decrease in circulating B cells [38] and HT also reverses the age-related decrease in CD4 and B cell numbers, T cell proliferative capacity and the increase in NK cells numbers [38–40].

However, the impact of HT on the response to infection or vaccination remains poorly characterized. A mouse model of menopause showed a significant reduction of influenza specific antibodies in ovariectomized mice; these levels were rescued to that of sham operated mice by the administration of estrogen [41]. This indicates that HT may boost vaccine responses in post-menopausal women. Since vaccination represents an important preventative health measure in older adults, and the role of HT is controversial, studies in this area have significant public health impact. In this study, we investigated the impact of menopause and estrogen therapy (ET) on lymphocyte frequency and the immune response to seasonal influenza vaccine in healthy hysterectomized women. We compared values of these parameters to those observed in young women of reproductive age as well as post-menopausal women not receiving any form of HT.

## Materials and Methods

### Subjects

The human subjects Institutional Review Board of Oregon Health and Science University approved all study procedures and documents. Every participant gave written consent before participation and research information was kept confidential. The study consisted of 3 arms: A) 15 young adults 19–38 years of age with normal menstrual cycles not currently taking contraceptives (Table 1); B) 15 women aged 55–63, at least two years post-menopausal and had not received hormone replacement therapy for at least 4 years (Table 2); C) 15 post-menopausal hysterectomized women aged 47 to 64 who were receiving estrogen therapy (Table 3). Women who were smokers, pregnant or breast-feeding, using hormonal birth control, on combined hormone replacement therapy, or using corticosteroids were excluded from the study. A 10ml heparinized blood sample was obtained on the day subjects received their seasonal flu shot (Day 0), one week (window 7–10 days) and one month (window 30–35 days) after immunization. Cycling women were asked to arrange their first visit to fall on one of the first 12 days of their cycle. Subjects received the seasonal trivalent inactivated influenza vaccine Fluzone (Sanofi Pasteur).

**Table 1:** Young Adult Women.

Subject ID	Age	Estrogen Levels			BMI (KG/m^2^)	History of smoking
		Visit 1	Visit 2	Visit 3		
1	23	117.0	40.8	47.3	25.2	N
2	24	31.9	118.0	59.2	19.5	N
4	35	98.9	172.0	57.5	20.5	Y
7	38	74.4	32.4	217.0	23.5	Y
9	19	37.4	< 20.0	72.1	25.0	N
15	36	39.0	< 20.0	30.1	24.8	N
16	29	108.0	101.0	69.9	27.0	N
19	26	124.0	23.9	74.0	21.0	N
20	35	115.0	21.0	135.0	21.4	N
21	24	109.0	56.0	81.7	25.5	N
22	22	21.2	45		22.5	N
23	28	< 20.0	< 20.0	97.3	21.5	Y
24	24	< 20.0	61.9	26.5	22.5	Y
27	25	50.1	39.7	64.0	20.0	N
29	33	228.0	50.9	57.2	21.6	Y
**Average**	28.1±5.9					

**Ethnicity:** Subject 9-Black, Subject 20-Asian, All other subjects in this group were White

**Table 2:** Post-menopausal (P.M.) Women no HT.

Subject ID	Age	Years P.M.	Previous hormone use and time since last dose (years)		BMI (KG/m^2^)	History of smoking
3	57	4	Never	NA	24.0	N
6	55	4	Never	NA	28.0	N
10	63	34	Combined	7	19.8	Y
11	56	8	Prempro	7	21.7	N
12	56	4	Never	NA	26.0	N
13	59	3	Never	NA	26.4	N
14	55	2	Never	NA	26.6	N
18	60	20	Combined	16	22.8	N
25	63	13	Combined	5	22.0	N
26	61	7	Never	NA	28.5	Y
30	56	14	Premarin	10	21.0	N
31	59	5	Climara	5	23.6	N
33	60	4	Estrodiol/Prometrium	4	24.2	Y
34	59	14	Prempro	5	23.4	N
37	61	12	Never	NA	21.6	Y
**Average**	58.7±2.7					

**Ethnicity:** All subjects in this group were White. NA indicates hormonal therapy was never received.

**Table 3:** Post-menopausal (P.M.) Women receiving ET.

Subject ID	Age	Years P.M.	ET regimen and length of time on regimen (years)		Estrogen Levels (pg/ml)			BMI (KG/m^2^)	History of smoking
					Visit 1	Visit 2	Visit 3		
8	61	25	Premarin (0.625mg)	25	<20.0	<20.0	<20.0	22.5	N
17	57	34	Estrace (2mg)	34	158	200	185	23.8	N
28	58	3	Vagifem (25mg)	2	<20.0	<20.0	<20.0	26.0	N
35	61	30	Ogen (0.6mg)	20	29.3	39.5	30.0	26.3	N
36	57	9	Estrogel (1.25mg)	5	47.5	34.9	41.2	23.1	N
38	59	5	Estradiol (0.5mg)	5	57.2	60.6	46.0	28.1	Y
39	47	11	Estrace (0.5mg)	5	60.9	64.0	54.9	29.1	N
40	64	19	Estratest HS	17 or 7*	29.7	<20.0	32.9	26.4	N
42	61	11	Vivelle Dot (0.05mg)	11	37.3	46.1	36.4	25.8	N
43	64	12	Vivelle Dot (0.05mg)	12	44.9	<20.0	32.0	26.0	N
44	57	8	Estrace (0.5mg)	4	29.3	28.5	30.1	23.8	N
45	56	19	Vivelle Dot (0.05mg)	2	32.3	23.6	43.0	29.2	N
46	64	7	Premarin (0.3mg)	7	36.6	38.6	56.1	28.8	N
47	55	6	Femring (0.05mg)	5	26.8	27.2	24.8	25.1	N
48	55	23	Premarin (0.45mg)	5	<20.0	24.1	22.5	25.6	N
Average	58.4±4.5								

**Ethnicity:** All subjects in this group were White.

*Participant 40 also participated in the WHI and it is unknown whether she received placebo or Estrogen.

### Blood sample analysis

Complete blood cell counts (CBCs) were obtained with a Hemavet complete blood count machine (Drew Scientific, Waterbury, CT). Blood samples were centrifuged over a ficoll density gradient in order to isolate peripheral blood mononuclear cells (PBMC) and plasma.

### Determination of plasma hormone levels

Serum concentrations of estradiol-17β (E2) and progesterone (P4) were determined by the Endocrine Technology and Support (ETS) Core at the Oregon National Primate Research Center (http://www.ohsu.edu/xd/research/centers-institutes/onprc/research-services/research-support/endocrine-technology.cfm) using an Immulite 2000 (Siemens Healthcare Diagnostics, Deerfield, IL). The sensitivity of the E2 and P4 assay by the Immulite 2000 is 20 pg/ml and 0.2 ng/ml, respectively. The E2 antibody used by the Immulite 2000 platform does not cross-react with Equilin, the main estrogen component within Premarin, alpha Equilenin, Ethiny-Estradiol, and 3- or 17-substrates of sulfate, valerate or propionate.

### Determination of T and B cell numbers and subsets

To measure T and B cell numbers, PBMCs were stained with anti- CD20 (eBioscience, San Diego, CA), CD8b (Beckman Coulter, Miami, FL), CD4, CD28, CD95, and CD27 (BioLegend, San Diego, CA) antibodies. Naïve and memory CD4 and CD8 T cells subsets were determined as previously described [42]. Naïve T cells were identified as CD28+CD95-, central memory as CD28+CD95+ and effector memory as CD28-CD95+. Memory B cells were identified based on the expression of CD27 [43]. The samples were acquired using an LSRII flow cytometer (Beckton Dickenson, San Jose, CA) and the data were analyzed using FlowJo software (TreeStar, Ashland, OR). The percentages of T cell subsets were converted to absolute numbers per μL using lymphocyte numbers from corresponding whole blood CBCs.

### Human IL-6 enzyme linked immuno-sorbant assay

IL-6 ELISA were performed using an ‘UltraSensitive’ Human IL-6 ELISA kit (Invitrogen, Camarillo, CA) following manufacturers instructions. Briefly, 100ul of sample serum was added in duplicate to precoated plates and incubated for 3 hours at 37°C. Plates were washed six times before the addition of 100ul of biotin conjugate per well, which was then incubated for 45 minutes at room temperature. Plates were again washed six times before a second 45 min incubation at room temperature with 100ul of streptavidin-HRP per well. After another six washes, plates were incubated at room temperature with 100ul of stabilized chromogen and the reaction stopped after 30 minutes with 100ul of stop solution per well. Color development was then read on a Spectramax 190 (Molecular Devices, Sunnyvale, CA) microplate reader at 450nm. Each plate contained manufacturer provided standards against which sample concentrations were determined, as well as an in house control.

### Measuring antibody responses

IgG end point titers were determined by standard ELISA using plates coated with the same Influenza Virus Vaccine (Fluzone, Sanofi Pasteur) that subjects received. After blocking and washing, threefold dilutions of plasma were added in triplicates. Bound IgG were visualized by the addition of HRP conjugated goat anti-human IgG (Open Biosystems, Huntsville, AL) and OPD substrate. The optical density was measured at 490 nm using an ELISA plate reader (SpectraMax 190, Molecular Devices, Sunnyvale, CA). Endpoint titers were determined by linear regression following log-log transformation and using 0.1 as the cut off and then normalized to a control sample included on each plate.

### Measuring frequency of influenza specific T cells

PBMCs from each subject were either untreated (negative control), stimulated with influenza virus PR8 overnight at 37°C in the presence of brefeldin A (Sigma, St Louis, MO), or stimulated for 6hr with anti-CD3 in the presence of brefeldin A (positive control). At the end of the incubation, PBMCs were stained with anti-CD8β (Beckman Coulter) and CD4 (BioLegend) antibodies. The cells were then fixed and permeabilized to allow for intracellular staining with anti-IFNγ and TNFα antibodies (Biolegend). Fluorescence was measured on an LSRII flow cytometer and the data analyzed using FlowJo software.

### Statistical analysis

Statistical analysis was performed using the SAS Software version 9.2 (SAS Institute Inc., USA) and Statistica (StatSoft, Tulsa, OK). Repeated measures ANOVA followed by Tukey post-hoc comparisons of means was used. Bayesian information comparison was used to determine the optimal correlation within subject. P values were adjusted by BMI and past smoking history.

## Results

### Study Population

A total of 45 subjects were enrolled (15 in each group), and all subjects completed the study. All young adult women were White except for 2 individuals (Black and Asian), had an average age of 28.1± 5.9, and BMI of 22.8± 2.3 (Table 1). All subjects within group B (post menopausal women not currently receiving HT) were White with an average age of 58.7±2.7 and BMI of 24±2.6. Approximately 50% of the women in Group B previously used HT (Table 2) but all women discontinued it at least 4 years prior to enrolling in this study. Subjects in group C (hysterectomized women receiving ET) were White with an average age of 58.4±4.5 and BMI of 26±2.1, and used different ET products summarized in Table 3. Plasma E2 levels were measured at each visit for all subjects. As expected, the levels of variation in plasma E2 levels between subject and visit were highest in young adult women (30pg/ml—100 pg/ml E2, Group A, Table 1). Plasma E2 levels also showed considerable variation in women receiving ET (Table 2). As expected, post-menopausal women not currently receiving HT had no detectable E2 levels. None of the subjects were current smokers, although 4 women, 5 women and 1 women in groups A, B and C respectively had a previous history of smoking.

### Menopause and ET impact on lymphocyte subset distribution

**Fig 1:**
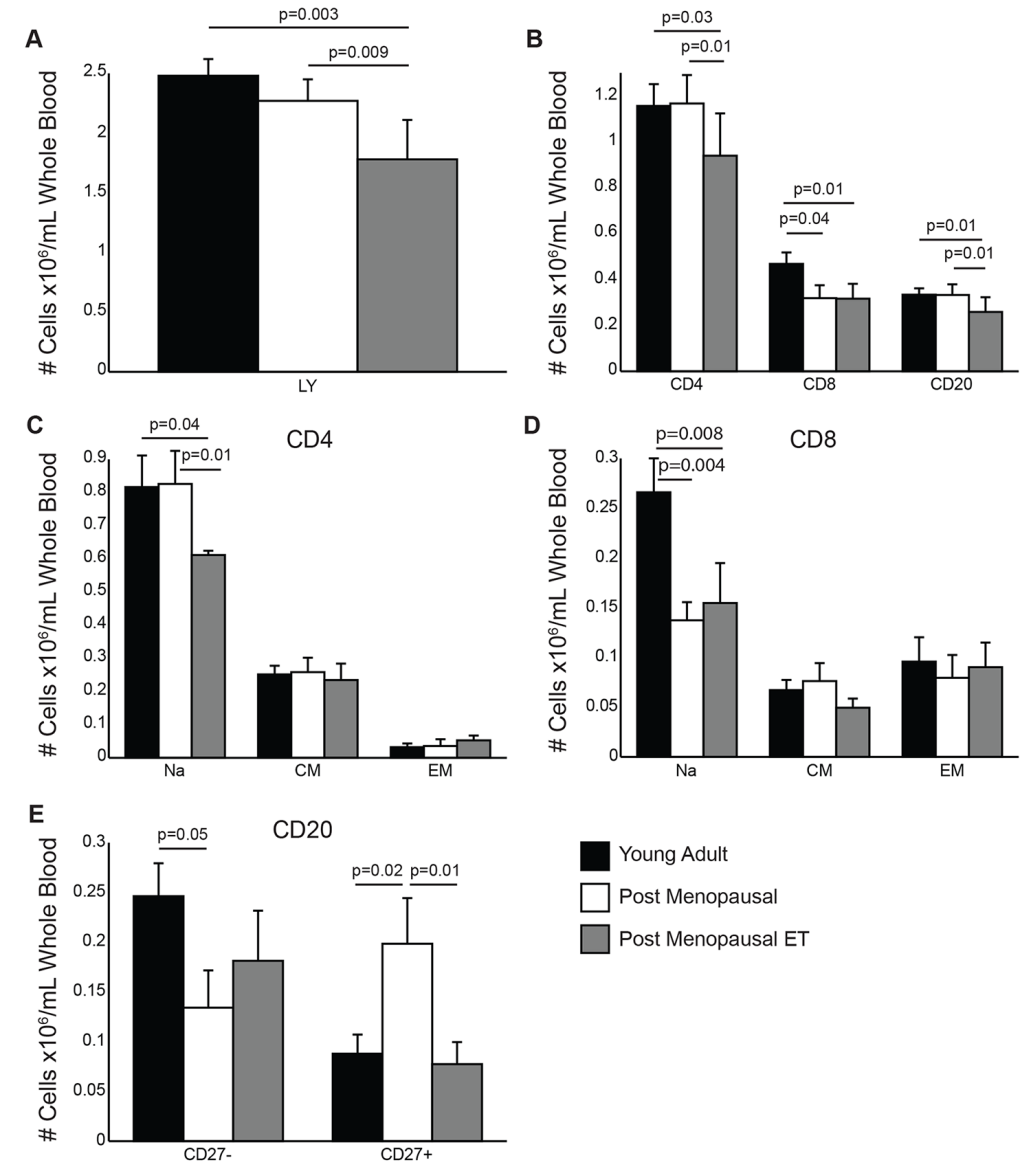
Impact of menopause and ET on lymphocyte frequencies. (A) The number of lymphocytes on visit 1 /μl blood based on complete blood cell counts (CBC) values. (B) Frequency of CD4 T cells, CD8 T cells and CD20 B cells was determined in PBMC using flow cytometry (FCM). The percentages were then converted to absolute numbers of cells/μl blood using the lymphocyte counts obtained from the CBCs. (C) Frequency of naïve (Na), central memory (CM) and effector memory (EM) CD4 T cells was determined using FCM and were then converted to number/μl of blood using the CD4 numbers obtained earlier. (D) Numbers of Na, CM and EM CD8 T cells was determined as described for CD4 T cells. (E) Frequencies of naïve (CD27-) and memory (CD27+) B cells were determined by FCM and then converted to absolute numbers of cells/μl blood using the CD20 numbers obtained earlier.

Aging results in significant changes in lymphocyte subset frequencies [44–46]. Therefore, we investigated the impact of menopause and ET on lymphocyte numbers and subset distribution (Fig 1). Our data shows that hysterectomized women receiving ET have lower lymphocyte numbers than young adult women and post-menopausal women not receiving HT (Fig 1A). In line with this observation, the number of CD4 and CD20 B cells was also reduced in hysterectomized women receiving ET compared to post-menopausal women not receiving HT (Fig 1B). Interestingly, the number of CD8 T cells was significantly lower in both post-menopausal women groups compared to young adult women (Fig 1B).

We next investigated the impact of menopause and ET on naïve and memory lymphocyte subset distribution. The number of naïve CD4 T cells was reduced only in hysterectomized women receiving ET compared to young adult women (Fig 1C). This difference in frequency of naïve CD4 T cells accounted for the decreased frequency of total CD4 T cells in post-menopausal women receiving ET. On the other hand and as described for total CD8 T cell numbers, we detected a significant decrease in the frequency of naïve CD8 T cells in both groups of post-menopausal women compared to adult women (Fig 1D). In contrast to these observations, we detected a decreased frequency of naïve B cells (CD27-) and a concomitant increase in the frequency of CD27+ memory B cells in post-menopausal women not receiving HT compared to young adult women (Fig 1E), whereas the numbers of naïve and memory B cells in post-menopausal women receiving ET were comparable to those in young adult women (Fig 1E).

### Menopause and ET modulate inflammatory cytokine production

**Fig 2:**
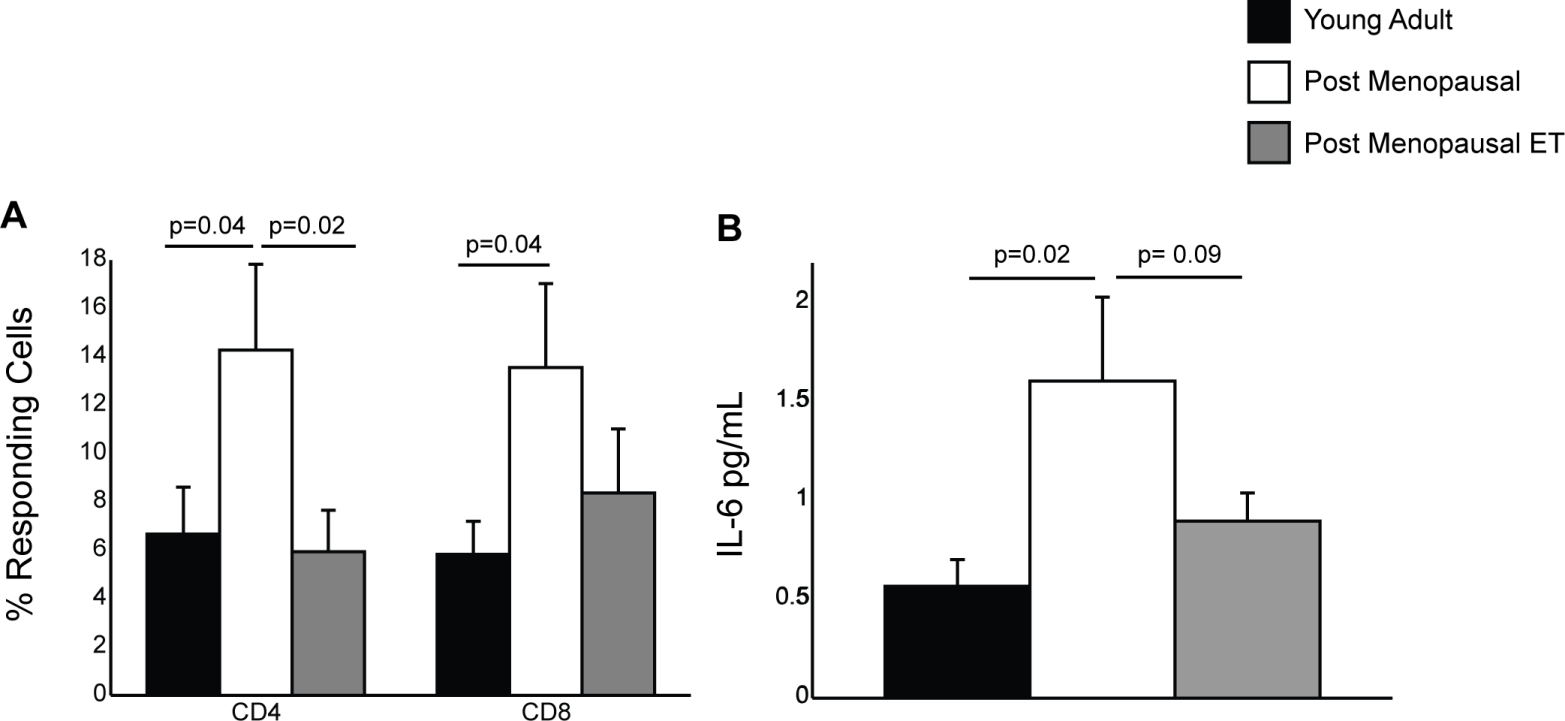
Impact of menopause and ET on IL-6 levels and TNFα and IFNγ production. (A) Frequency of CD4 and CD8 T cells that secrete TNFα, IFNγ or both in response to CD3 stimulation was determined by intracellular cytokine staining and FCM using PBMC collected during visit 1 before the administration of the influenza vaccine. (B) Plasma IL-6 levels using samples collected during visit 1 were determined by ELISA.

Aging is associated with increased production of pro-inflammatory cytokines by T cells [47,48]. Therefore, we measured inflammatory cytokine production by T cells in response to polyclonal CD3 stimulation using PBMC collected during visit 1 (Fig 2A). Specifically, we measured the frequency of CD4 and CD8 T cells that produced TNFα, IFNγ, or both using intracellular cytokine staining. We detected increased frequency of CD4 and CD8 T cells producing TNFα, IFNγ or both in post-menopausal women not receiving HT compared to young adult women consistent with previous studies comparing cytokine production by T cells collected from young and old subjects [47,49,50]. In contrast, frequencies of CD4 and CD8 T cells producing TNFα, IFNγ or both were comparable in post-menopausal women receiving ET (group C) and young adult women (group A) (Fig 2A).

Aging is also associated with increased circulating levels of inflammatory cytokines, notably IL-6 and CRP, often referred to as inflammaging [44,48,51,52]. Thus, we next measured plasma levels of the key inflammatory cytokine IL-6 during the first visit (prior to vaccination). Our analysis revealed that post-menopausal women not receiving HT had a significantly higher level of plasma IL-6 than young women (Fig 2B). Plasma levels of IL-6 were previously shown to be lower in post-menopausal women receiving unopposed estrogen compared to women not receiving HT [36,53]. Although not statistically significant, we also detected a clear trend towards lower plasma IL-6 levels in the ET group compared to non-HT group (p = 0.09).

### The immune response to seasonal influenza vaccine

**Fig 3:**
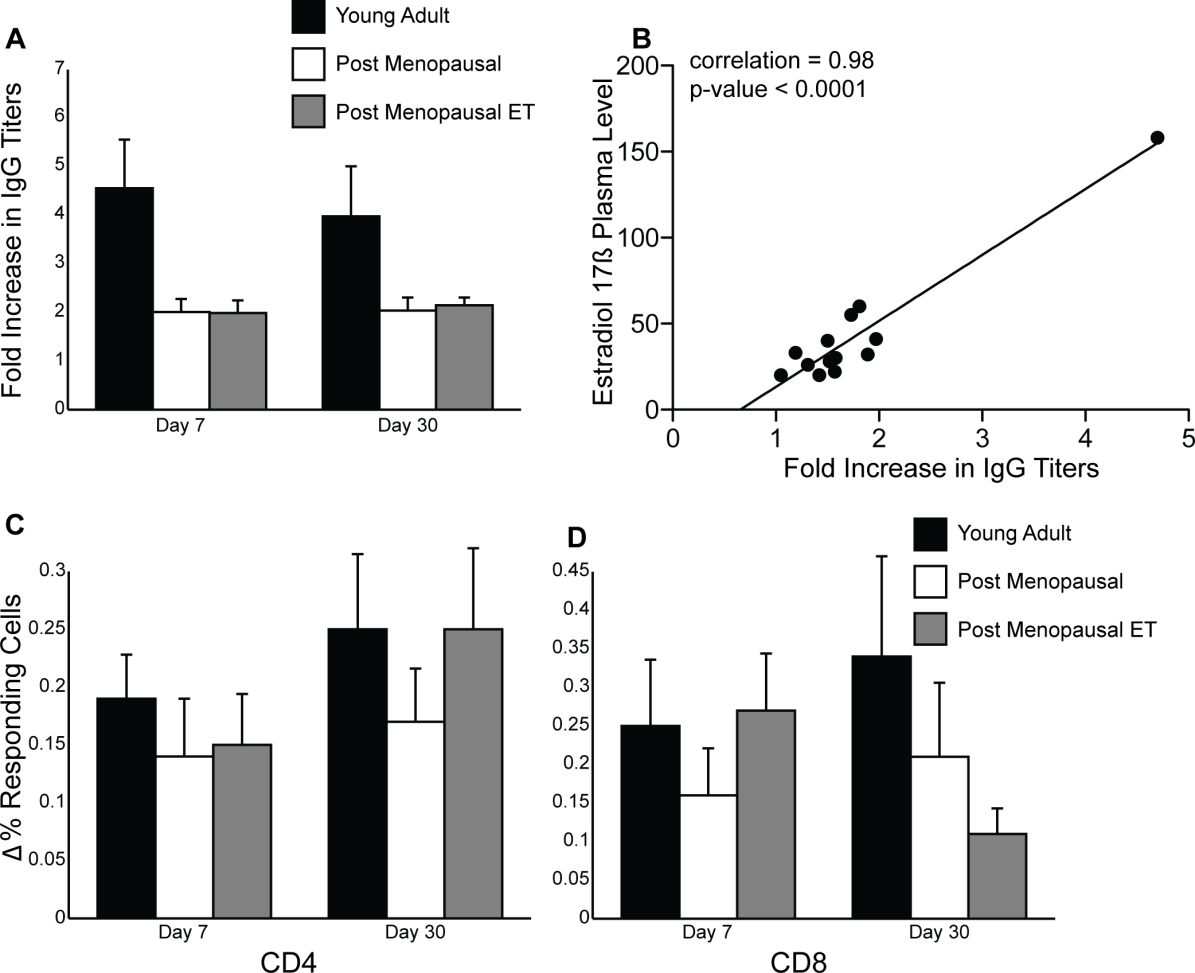
Impact of menopause and ET on immune response to seasonal influenza vaccine. (A) IgG end point titers were determined using standard ELISA and then the fold increase over baseline was calculated for visits 2 and 3. (B) Correlation between plasma E2 levels on visit 1 (day of vaccination) and fold increase in IgG titer during visit 2 was calculated for women in group C. (C) Frequency of flu-specific CD4 T cells was measured using intracellular cytokine staining and flow cytometry. The increased frequency of responding T cells was determined by subtracting the values obtained on visits 2 and 3 from those obtained on visit 1. (D) Same analysis was carried out for CD8 T cells.

Previous studies have shown that hemagglutination inhibition (HI) assays are not a good correlate of vaccine efficacy in the elderly [54]. Therefore, we measured the fold increase in IgG titers in response to influenza vaccination by standard ELISA (Fig 3A). IgG titers increased in all three groups at day 7 post-vaccination and remained stable at day 30 as previously reported for adults receiving the trivalent inactivated influenza vaccine [55] As expected, young adult women generated a robust antibody response compared to older post-menopausal women. There was no difference in the fold increase in IgG titers between the two post-menopausal groups, but given the differences in E2 plasma levels, we refined the analysis by measuring the correlation coefficient between plasma E2 levels on the day of vaccination and IgG fold increase on day 7 post-vaccination in post-menopausal women receiving ET (Fig 3B). Our analysis revealed a positive correlation of 0.98 with a p value <0.0001 within group C. This correlation remained significant after removing the subject with the highest E2 plasma levels (correlation = 0.59; p-value = 0.0439). This correlation remained significant on day 30 (correlation = 0.77; p-value = 0.0008) but once the subject with highest plasma E2 levels was omitted, the correlation was no longer significant (correlation = 0.32; p-value = 0.2634). Additionally, we measured the correlation coefficient between plasma E2 levels on the day of vaccination and IgG fold increase on days 7 and 30 post-vaccination in young adult women and saw no correlation (slope <0.01 and p>0.9 in both cases). These data suggest that plasma E2 levels may only play a role in post-menopausal women.

We next evaluated the impact of ET on the T cell response to the seasonal influenza vaccine (Fig 3). Following vaccination all subjects showed an increase in the frequency of influenza-specific CD4 and CD8 T cells on both day 7 and 30 post vaccination. There were no statistically significant differences in the increase in T cell responses between all three groups. On day 30 post vaccination, we detected a trend indicating a further increase in CD4 T cell response in young adult women and post-menopausal women receiving ET but not in post-menopausal women not receiving HT (Fig 3C). In contrast, CD8 T cell response in post-menopausal women receiving ET on day 30 post vaccination are slightly reduced compared to day 7 (Fig 3D).

## Discussion

Aging is associated with a general dysregulation of the immune system referred to as immune senescence that leads to diminished capacity to respond to infection and vaccination [23]. Since several studies have shown that ovarian steroids modulate immune response in women [56–58], menopause-associated loss of ovarian steroids is likely to have a significant impact on immune senescence. Indeed, both clinical and experimental observations suggest that the loss of ovarian steroids correlates with several immunosenescent changes that are in turn modulated by hormone therapy (HT) [35,40]. However, the impact of HT on lymphocyte homeostasis and the immune response to vaccination and/or infection remains poorly understood. In this study we specifically investigated the impact of estrogen therapy (ET) on T and B cell subset distribution and function.

We report that post-menopausal women receiving ET have reduced lymphocyte numbers and that this reduction was due to a decrease in CD4 T and B cell numbers. Unfortunately, we did not have access to longitudinal samples collected before and after hysterectomy to link this decrease to changes in ovarian steroid levels. It is possible that the loss in lymphocytes was mediated by the sudden decrease in levels of circulating ovarian steroids following hysterectomy. This is in line with previous studies where total abdominal hysterectomy and oopherectomy in adult women were shown to decrease the number of circulating B cells and CD4/CD8 ratio [38]. In contrast to our data, in these previous studies ET reversed the decrease in CD4 T and B cells [38,39]. There are several differences between the previous studies and the current one that can explain this discrepancy. While the previous studies examined young perimenopausal women that underwent a bilateral oopherctomy as well as a hysterectomy, 9/15 women in our study were over the age of 50 when they received a hysterectomy (Table 3). In addition, whereas all the women in the Kumuru study received ET within 30 days of their surgery, 7/15 women in our study did not initiate ET until several years after their surgery (Table 3). Finally, various types of ET were used in our study whereas all the subjects in the Kumuru study received transdermal E2. This current study is underpowered to examine the impact of age at hysterectomy and onset of ET and the type of ET on lymphocyte homeostasis but this will be the focus of future studies.

An alternative explanation for the decreased lymphocyte numbers is that ET interferes with lymphopoiesis given that puberty correlates with the onset of thymic involution [59]. The decreased frequency of naïve CD4 T cells observed in post-menopausal women receiving ET would support this hypothesis. In contrast to CD4 T cells, frequency of naïve CD8 T cells decreased in both groups of post-menopausal women compared to young adult women which suggests that aging has a more significant impact on loss of naïve CD8 T cells than menopause. However, the decreased frequency of CD4 T cells in post-menopausal women receiving ET did not impact the T cell response to influenza vaccination since we reported a comparable increase in the frequency of influenza T cells in all three groups.

Although post-menopausal women receiving ET had fewer B cells, we detected improved preservation of naïve B cells compared to post-menopausal women not receiving HT. Naïve lymphocytes are the body’s reserve to respond to novel pathogens that have not been previously encountered. Thus, increased frequency of these cells could confer advantage to the host in the face of infection. Interestingly, fold increase in IgG titers on day 7 post vaccination correlated significantly with plasma E2 levels measured on the day of vaccination in post-menopausal women receiving ET. These findings are in line with previous studies in rodents, which showed that E2 treatment of control or ovariectomized mice enhanced protection against HSV-2 challenge [32]. In those studies, although antibody titers in E2 treated mice were not significantly higher than those observed in untreated mice, the neutralization potential was significantly improved [32]. Our data are also in agreement with previous in vitro studies that showed increased IgG and IgM production by human PBMC after E2 stimulation [14]. It should be noted that the correlation between plasma E2 levels and vaccine-specific IgG titers was only significant for day 7 and only in post-menopausal women, suggesting that plasma E2 levels might be particularly important for older women. We measured no additional increase in IgG titers after day 7 post vaccination, which is in line with previous reports from adults vaccinated with live attenuated influenza viruses or a trivalent inactivated vaccine where serum IgG levels increased at 14 and remained stable at 28 days post vaccination [55]. On the other, ET had no effect on the T cell response to vaccination. However, the seasonal influenza vaccine is a fully inactivated vaccine that elicits primarily an antibody response, thus the induction of T cell responses is not usually substantial following vaccination.

Our data also showed that ET is associated with reduced production of inflammatory cytokines TNFα and IFNγ by T cells. Previous studies have reported similar findings with regards to natural killer cells [60] and T cells [61]. As previously described, we saw an increase in plasma IL-6 levels in post-menopausal women not receiving HT compared to young women [62]. Our data also suggests that ET can lower circulating IL-6 levels in post-menopausal women. This observation is in line with data from previous studies that showed that women receiving transdermal estrogen experienced a significant decrease in IL-6 serum levels after ET initiation [36] and that serum IL-6 levels show a negative correlation within users [36] and in women spanning the transitional stages of menopause aged 40 to 65 years [63]. Since the age-related increase in inflammation has been correlated with increased incidence/severity of chronic diseases, these observations albeit modest, suggest that ET can reduce inflammaging, which in turn could ameliorate inflammatory diseases such as osteoporosis and cardiovascular disease [64,65]. Moreover, previous studies found a positive correlation between plasma IL-6 levels and severity of symptoms [66], as well as exacerbation of disease and lung damage [67] in influenza infected patients. Therefore, the lower plasma levels of IL-6 in post-menopausal women receiving ET could potentially result in better outcomes after influenza infection compared to post-menopausal women not receiving HT.

In summary, although hysterecotmized post-menopausal women receiving ET had a decreased number of circulating lymphocytes, they exhibited better preservation of naïve B cells, lower levels of inflammatory cytokine production by T cells, and slightly reduced levels of circulating IL-6 levels. We further show that the decreased number of T cells did not negatively impact their ability to generate a T cell response following seasonal influenza vaccination and that higher plasma E2 levels correlate with increased antibody IgG titers to influenza vaccine early after vaccination. This is a small cohort and our observations need to be validated in a larger study. Nevertheless, given the benefits conferred by ET on bone density, cognitive function and cardiovascular disease [68], the studies presented here emphasize the need for additional studies to elucidate the optimum route of E2 delivery and in particular the effect of oral (subject to hepatic first pass effects) versus transdermal (including vaginal) administration. They also highlight the need to identify a target blood level of E2 needed to enhance immune response to vaccination in post-menopausal women and achieve a better understanding of the impact of acute and chronic exposure to estrogen on immunity.

## Supporting Information

S1 File All Original Data. (XLSX)
